# Characteristics of Reconstituted Collagen Fibers from Chicken Keel Cartilage Depends on Salt Type for Removal of Proteoglycans

**DOI:** 10.3390/molecules26123538

**Published:** 2021-06-10

**Authors:** Anna Pudło, Szymon Juchniewicz, Wiesław Kopeć

**Affiliations:** Department of Functional Food Products Development, Faculty of Biotechnology and Food Sciences, Wrocław University of Environmental and Life Sciences, 37 Chelmonskiego Str., 51-650 Wrocław, Poland; juchniewiczszymon@gmail.com (S.J.); wieslaw.kopec@upwr.edu.pl (W.K.)

**Keywords:** cartilage, collagen type II, reconstituted fibers, pepsin, proteoglycans

## Abstract

The aim of the presented research was to obtain reconstituted atelocollagen fibers after extraction from poultry cartilage using the pepsin-acidic method in order to remove telopeptides from the tropocollagen. Firstly, we examined the extraction of collagen from the cartilage extracellular matrix (ECM) after proteoglycans (PG) had been removed by the action of salts, i.e., NaCl or chaotropic MgCl_2_. Additionally, the effects of the salt type used for PG and hyaluronic acid removal on the properties of self-assembled fibers in solutions at pH 7.4 and freeze-dried matrices were investigated. The basic features of the obtained fibers were characterized, including thermal properties using scanning calorimetry, rheological properties using dynamic oscillatory rheometry, and the structure by scanning electron microscopy. The fibers obtained after PG removal with both analyzed types of salts had similar thermal denaturation characteristics. However, the fibers after PG removal with NaCl, in contrast to those obtained after MgCl_2_ treatment, showed different rheological properties during gelatinization and smaller diameter size. Moreover, the degree of fibrillogenesis of collagens after NaCl treatment was complete compared to that with MgCl_2_, which was only partial (70%). The structures of fibers after lyophilization were fundamentally different. The matrices obtained after NaCl pretreatment form regular scaffolds in contrast to the thin, surface structures of the cartilage matrix after proteoglycans removal using MgCl_2_.

## 1. Introduction

Reconstituted collagen fibers (self-assembled after increasing the ionic strength or pH in monomer solutions) is the basis for creating matrices and scaffolding of biomaterials replacing soft or hard tissues, e.g., bones [1,2,3,4,5]. The main materials used for this purpose are reconstituted fibers of type I collagen obtained by the acid method or acid-enzymatic method with pepsin [6]. The latter is considered the most advantageous, as it removes telopeptides (the so-called atelocollagen is then formed), which reduces the immunogenicity of the obtained biomaterials that can be introduced into human tissues [7,8,9]. Among the many types of collagen, type II collagen is of particular interest, as it builds cartilage extracellular matrix (ECM) together with proteoglycans (PG) (mainly composed of sulfated glycosaminoglycans (GAG) linked to hyaluronic acid chain) [10]. Type II collagen is a raw material used in the production of dietary supplements; it is mainly made of by-products from the processing of fish cartilage. Another by-product that has been employed to some extent is poultry cartilage (keel) obtained from skeleton after muscle cutting. In addition to using collagen II as a dietary supplement (mainly as hydrolysate), reconstituted collagen II scaffolds are applied as biomaterial to replace cartilage [2,3,4,5]. Contrary to the well-known structure of reconstituted collagen fibers, mainly type I, collagen II fibers are less recognized. Hence, the basic thermal and rheological properties of reconstituted collagen II fibers are not well known. The procedure for the extraction and purification of collagen from cartilage, including the separation of the proteoglycan fraction, is of fundamental importance for the characteristics of these fibers. It is assumed that to obtain low immunogenicity of atelocollagen and high reconstitution capacity under conditions of increased ionic strength or pH, the acid-pepsin procedure should be utilized, where two possible extraction methods can be employed. The first is direct degradation of the cartilage in acidic solution by pepsin, whereas the second involves initial separation of the proteoglycan fraction followed by collagen extraction. The first method produces atelocollagen extract high in proteoglycan contamination. In contrast, the second method provides cleaner collagen and facilitates the use of valuable proteoglycan fraction (mainly glycosaminoglycan chondroitin sulfate) for dietary supplements or the bioengineering of matrix components [2,6].

Classic procedures usually involve the initial separation of proteoglycans, which is relatively easily achieved using buffered NaCl solution (so-called non-denaturing conditions) or chaotropic compounds, i.e., guanidine (most often a hydrochloride) or magnesium chloride (MgCl_2_) on the G1 domain of the core protein and protein-linked proteoglycans to the chain of hyaluronic acid [11]. Such procedures enable the preservation of the collagen matrix of ECM in its entirety. Since guanidine cannot be used in food preparations (dietary or food supplements), it is advantageous to dissociate proteoglycans in NaCl or MgCl_2_ solution, as their possible residues do not preclude the use of proteoglycans in food. It is also beneficial in the extraction of collagen, as most procedures involve the precipitation of reconstituted collagen in saline solutions. Although the influence of non-denaturing conditions (NaCl) or chaotropic salts (MgCl_2_) on the dissociation of the proteoglycan fraction from the cartilage has been previously investigated, to the best of our knowledge, no data are available on the impact of these methods on the further course of isolation and structures obtained after the reconstitution of atelocollagen. Therefore, the aim of this study is to obtain reconstituted collagen II fibers from poultry cartilage using a method consisting of prior separation of proteoglycans as a result of the action of salts, i.e., NaCl or chaotropic MgCl_2_, which is followed by fiber structure characterization (also freeze-dried forms) as well as thermal and rheological features that are important for the formation of biomedical materials.

## 2. Results and Discussion

### 2.1. Chemical Composition of the Raw Material

The characteristics of cartilage indicate its suitability for obtaining purified forms of collagen useful in biomedical and food applications. The determined chemical composition of cartilage, i.e., dry matter, total protein, ash, collagen (hydroxyproline content), uronic acid, and glycosaminoglycans are presented in Table 1. The obtained results show that 8.50% of total protein is in the raw material; however, Shin et al. [12] reported a value of approximately 11.8% of total protein, but it had an approximately 4% higher dry matter level than that in our research (i.e., 13.10%). The differences result probably from the age of the chickens (very young 5-week-old broilers were studied, whereas in other studies, the age was over 7 weeks [13]). However, there is no difference in the share of ash per dry matter between our own and other studies [12,14]; this value is low and amounts to about 7%, indicating that the cartilage is not mineralized. In fish and reptile cartilage tissues, the total protein level is higher than in poultry cartilage, ranging from 12% to 15%, which is mainly due to the higher dry matter content of 25–45% [15,16]. However, the proportion of proteins (collagen) and carbohydrates in dry matter in most of the raw materials was similar or lower than in chicken keel cartilage (64.30% protein and almost 30% GAG in dry matter). 

Among collagen-rich poultry raw materials, chicken keel cartilage contains the highest amount of uronic acid. Nakano et al. [17] determined the value of 87.7 μg/mg dry weight for this raw material, while our own research showed an even higher level, i.e., 107 μg/mg dry weight (Table 1). Therefore, this raw material is potentially very valuable in terms of obtaining two main fractions, i.e., collagen and GAG. However, when generating pure forms of collagen, e.g., the reconstituted fibers, the necessity of removing large amounts of proteoglycans and their components, i.e., GAGs must be considered. One of the possibilities to remove GAGs is to use the chaotropic effect of salts; another is to use anion exchange column chromatography [18]. The purification and separation of collagen using column chromatography is also a long-established technique [19].

### 2.2. PG Extraction from Cartilage (Pretreatment of Cartilage)

The removal of proteoglycans from the cartilage during pretreatment in NaCl or MgCl_2_ salt solutions can be indicated through determination of the degree of their extraction from the chicken keel cartilage, which is expressed as the recovery of uronic acid and GAG in the solution in relation to the raw material (Table 2). The results show that 9M solutions of MgCl_2_ and buffered NaCl solution are equally effective in PG extraction from cartilage. The degree of proteoglycan extraction, especially after 48 h (23 °C), is very high and accounted for over 97% of the total amount in the starting material. 

### 2.3. Extraction of Pepsin-Solubilized Collagen—Reconstituted Fibers

After pretreatment of cartilage to remove PG, the precipitated collagen matrix was treated with pepsin (pH 2.0, citric acid). When 400 mg of enzyme for 1 g of raw material was used, a practically complete dissolution of collagen was observed (95–97% of hydroxyproline was found in solution), regardless of salt type used for pretreatment (Table 3). However, it should be emphasized that the amount of pepsin used in collagen extraction and raw materials rich in collagen was quite large, because for poultry or fish skins, smaller amounts of enzyme are to be used, e.g., about 100 mg/g [8,20,21]. Additional enzyme (300 mg/g) was used in the process of collagen recovery from broiler legs [22], and for mineralized tissues such as bone elements, it was even over 1000 mg/g [23].

Significant differences were found in the amount of obtained fibers depending on the method of proteoglycan removal (Table 3). The recovery of atelocollagen obtained in the form of reconstituted fibers from cartilage treated with NaCl-buffered solution (TRIS-HCl) reached approximately 95%, which was higher than that of the raw material treated with MgCl_2_ solution (approximately 71%). In reported studies, comparable high rates of collagen recovery from solutions (85–93%) in the form of fibers after washing the cartilage with NaCl solution were observed. Other authors obtained similarly high results only for the fibrillogenesis of type I collagen from fish skins [24,25]. A greater degree of recovery of collagen fibers after NaCl pretreatment of cartilage also resulted in a greater mass of the lyophilized fiber matrix per raw material (Table 3).

### 2.4. Differential Scanning Calorimetry (DSC)

As shown in Table 4, DSC thermograms of cartilage and fibers display a single endothermic transition (T_m_), which is the unwinding of the triple helix of collagen [26]. Pretreatment using NaCl solution did not significantly change T_m_ (65 °C vs. 64.5 °C for raw cartilage), but the removal of PG fraction with MgCl_2_ slightly lowers (62.5 °C) collagen denaturation temperature in the residue. The T_m_ of atelocollagen fibers obtained by the enzymatic-acid method (as a result of in vitro fibrillogenesis) was much lower than collagen in the cartilage (Table 4).

**Figure 1:**
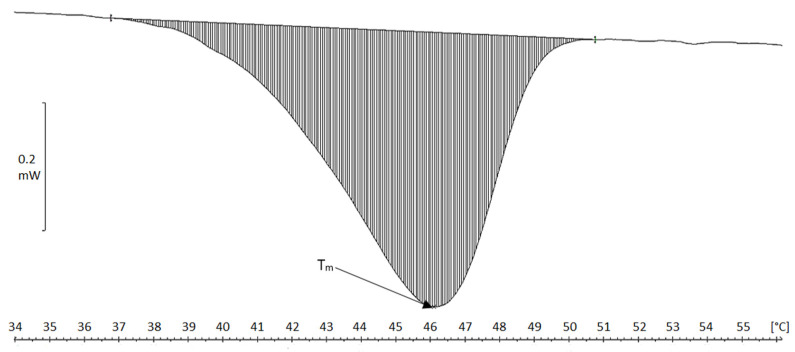
DSC thermogram of atelocollagen fibers in acid solution (obtained after pretreatment of the raw material in NaCl solution).

**Figure 2:**
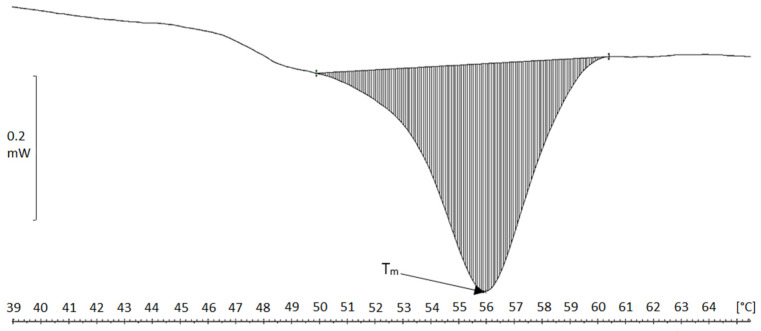
DSC thermogram of atelocollagen fibers in neutral solution (obtained after pretreatment of the raw material in MgCl_2_ solution).

Collagen fibers precipitated in acid solution were characterized by T_m_ of 45.5 °C and 46 °C, and a single transformation occurred in the thermograms in the temperature range 37–51 °C (Figure 1). The fibers obtained in neutral conditions by the dialysis of collagen solution against a phosphate buffer showed higher transformation temperatures T_m_, which were close to 56 °C (transition range 52–60 °C) (Figure 2), thus indicating higher thermal resistance in contrast to that found for acidic fibers. The production of collagen neutral fibers with higher thermal stability is important, as it facilitates the drying or freeze-drying processes (higher temperatures treatment) as well as the use of atelocollagen fibers to create matrices for bioengineering applications [27].

There were significant differences in the value of enthalpy change between the raw material, i.e., cartilage, the residue after proteoglycan removal (i.e., mainly the collagen matrix of cartilage), and reconstituted collagen fibers (Table 4). If ΔH is converted to the mass unit of collagen, then raw cartilage shows lower enthalpy than the residue after the removal of proteoglycans. However, the highest values were found for the reconstituted fibrils. Holmes et al. [28] showed that fibrillogenesis causes an increase in ΔH value for atelocollagen fibers of approximately 20%, and in our study, it was considerably higher—by four times—compared to tropocollagen in cartilage and amounted to about 100 J/g. Even higher values were found for fibers after reconstitution in neutral solution 156 J/g of collagen (after removal of PG in NaCl solution) and about 219 J/g of collagen (after removal of PG in MgCl_2_ solution). This corresponds to the relationship given by Singh et al. [29], who indicated that under acidic conditions, collagen structure is destabilized due to cleavage of hydrogen bonds, which caused lowering both T_m_ and enthalpy.

### 2.5. Rheological Properties of Reconstituted Collagen 

**Figure 3:**
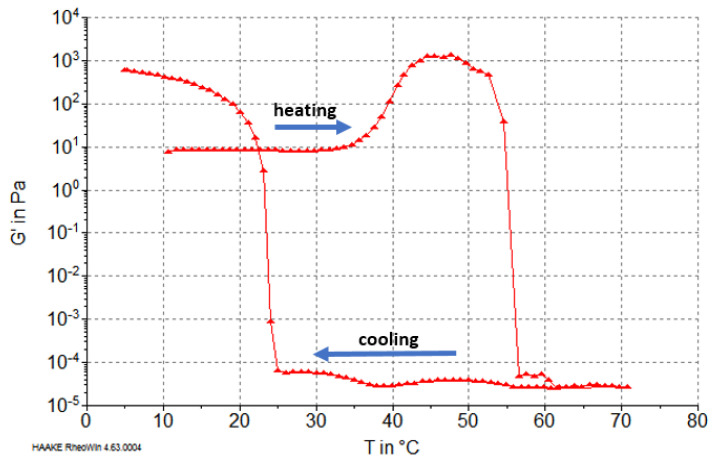
Changes in the G′ value of atelocollagen fibers (from cartilage after pretreatment with NaCl).

Characteristics of phase transformations of reconstituted collagen fibers during heating and cooling were determined based on the storage modulus G′ value (characterizing elastic properties of the material). The results showed that G′ was dependent on the type of raw material pretreatment (PG extraction). In the case of collagen after treatment in NaCl solution, during heating above 32 °C, an increase in G′ value was observed (Figure 3). This transition, although dependent on temperature change, was similar to the aggregation of collagen microfibrils to create gel at temperatures of 32–37 °C, i.e., below the denaturation temperature according to Forgacs et al. [30] and Yang et al. [31]. An increase of elastic properties and G′ value may also be caused by significant inhomogeneity in the gel structure, consisting of different concentrations of collagen fibrils at temperatures below the denaturation temperature (37 °C) [32]. Our results showed an increase of G′ before unfolding the collagen molecules in the range 37–46 °C (Figure 3). After that, at approximately 52 °C, the main phase transition was observed, which involved thermohydrolysis (gelatinization) and a related reduction of G′ to very low levels. This transformation completed at 56 °C (Figure 3). 

**Figure 4:**
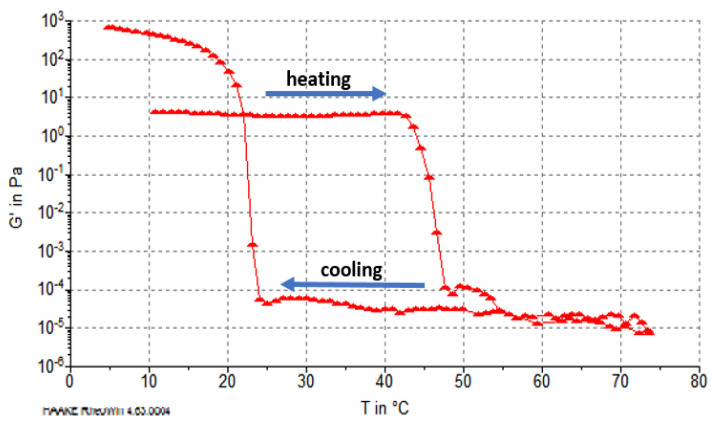
Changes in the G′ value of atelocollagen fibers (from cartilage after pretreatment with MgCl_2_).

During the cooling carried out after the heating phase, the collagen solution (actually gelatin) was gelled. This process began at approximately 25 °C and ended at approximately 8 °C. For fibers obtained from cartilage after pretreatment with MgCl_2_ solution, no increase in G′ was observed at temperatures below 40 °C, and the decrease of G′ (gelatinization) occurred at temperatures ranging from 42 to 48 °C; i.e., it was lower than for fibers obtained after treatment with NaCl solution (Figure 4). Therefore, a lower heat resistance of the reconstituted collagen was obtained after PG removal from the cartilage with MgCl_2_. 

However, it should be emphasized that the rheological characteristics of the gelatins of both types of collagen fibers (i.e., after heating to over 70 °C and gelatinization) were similar, and the sol–gel transformation during cooling began at 25 °C. The final G′ value after cooling for both types of gelatin was higher than that of the starting collagen fibers, which was related to the partial recovery of the triple helix upon cooling of the gelatin solutions, as reported by Eysturskard et al. [33].

### 2.6. Microstructure of Atelocollagen Fibers (Type II Collagen)

**Figure 5:**
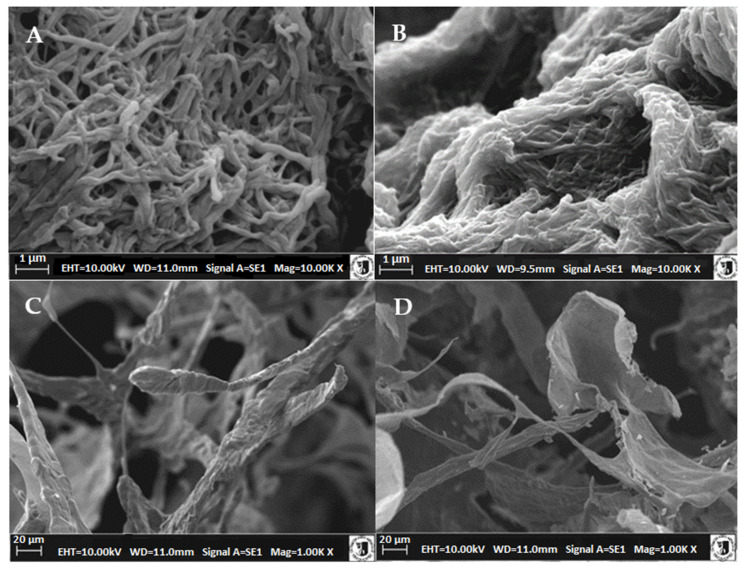
Electron microgram SEM of (**A**) reconstituted atelocollagen fibers (neutral solution at pH 7.4) isolated from chicken keel cartilage after preliminary proteoglycan extraction with NaCl solution; (**B**) isolated from chicken keel cartilage after extraction with MgCl2 solution; (**C**) freeze-dried preparations of reconstituted collagen fibers obtained in a neutral solution at pH 7.4; isolated from cartilage after preliminary extraction of proteoglycans with NaCl solution; (**D**) from cartilage after extraction of proteoglycans with MgCl2 solution.

Figure 5A,B show the undulated, intertwined fibrous structures formed by the basic units, the sub-fibers. We observed a different degree of aggregation of sub-fibers with a diameter of 0.1–0.2 µm (100–200 nm). These aggregates formed locally, weakly bound collagen fiber bundles (2–3 sub-fibers each) with a diameter of approximately 450 nm. The most contrasting were the SEM images of fibers obtained from collagen after PG removal with NaCl solutions. Upon extraction of PG from cartilage using MgCl_2_, the fiber structures were thicker and more blended (diameter 170–550 nm) (Figure 5B), or large fibrillar structures similar in size (1.4–3.6 µm) to collagen fiber bundles (longitudinal structures) in cartilage were formed. 

Type I, II, and III collagens can undergo fibrillogenesis [34], but only the process of type I collagen reconstitution was described in detail, including its structure [35]. The concentration of atelocollagen as well as the pH and ionic strength of their representative solutions are essential for the morphology and size of secondary fibrils. 

Filamentogenesis at low ionic strength and pH 7.0 led to the formation of collagen type I bundles of 100–200 nm in diameter [36]. Similar conditions were employed in our study, where the ionic strength of the solution with pH 7.4 was shaped only by 10 mM phosphate buffer, and the obtained collagen II fibers (sub-fibers) were characterized with a diameter of 100–200 nm. Typical reconstituted fibers of collagen type I derived from slaughter materials (skin or mammalian tendons) separated in solutions with high ionic strength reached 250–300 nm in diameter [37,38]. When it comes to type II collagen fibers, there are little data on them in the literature. For example, fish cartilage was previously used to obtain fibers with a diameter of 50–100 nm [24], while that from bovine cartilage was used to obtain fibers with a diameter of 80–120 nm [37]. No images of the reconstituted fibers of collagen type II from poultry cartilages are available in the literature. However, the morphological data of the folded fibers obtained in our study were similar to that presented by Liang et al. [16] for collagen type II from sturgeon cartilage.

The lyophilization of atelocollagen fibers leads to the formation of porous structures (matrices) containing protein fibers with diameters from approximately 5 to 20 μm with a partially preserved fiber structure (preparation after PG NaCl extraction) (Figure 5C) or thin-walled three-dimensional structures (preparation after PG MgCl_2_ extraction) (Figure 5D). These differences in structures obtained after lyophilization were unprecedented, as the thicker, more dense fibrils or filaments obtained from collagen after MgCl_2_ pretreatment gave thin, superficial structures resembling gelatin biomaterials. In contrast, the thinner fibers (after NaCl treatment) formed a very regular scaffold similar to those indicated by Pieper et al. [27], allowing the application of chondrocytes or structuring of surgical sponges.

The obtained ultrastructure images allow for interpretation of the previously discussed results. The thickness of the reconstituted collagen fibers obtained after MgCl_2_ pretreatment was considerably greater, and their dense structure indicated a different nature of the formed collagen fibers. This was due to variations in the initial procedures of removing proteoglycans from the cartilage. Of the two analyzed salts, MgCl_2_ showed a pronounced chaotropic effect that favors weaker water binding in the cartilage extracellular matrix after PG removal, which may alter the dissolution behavior of tropocollagen primary fibers. Although the efficiency of atelocollagen extraction from cartilage residue after PG removal in both salt solutions was comparable, the recovery of self-assembled collagen fibers was substantially different, i.e., higher for NaCl pretreated cartilage. This may be due to the extraction of larger collagen fragments from the cartilage residue or the higher degree of fiber/filament polymerization in solutions after MgCl_2_ pretreatment. For fibers pretreated with NaCl, the degree of polymerization may be lower (as indicated by thinner fibers after fibrillogenesis). Although the efficiency of atelocollagen extraction from cartilage debris after PG removal in both salt solutions was similar, the recovery of self-assembled collagen fibers was substantially different; i.e., it was higher for NaCl pretreated cartilage and lower for MgCl_2_. These differences persist also after dialysis and influence the rheological behavior of the fibers. Namely, collagen with thin fibers clearly shows gelation before thermal denaturation, whereas thick fibers do not gel at temperatures below the main transformation. However, these variations were not significant enough to affect the thermal properties of dialyzed fibers, although thicker fibers had slightly higher enthalpy of main conversion (gelatinization). Furthermore, large differences were observed after freeze drying, which was a technique used for fixing collagen matrices intended for biomedical applications.

**Figure 6:**
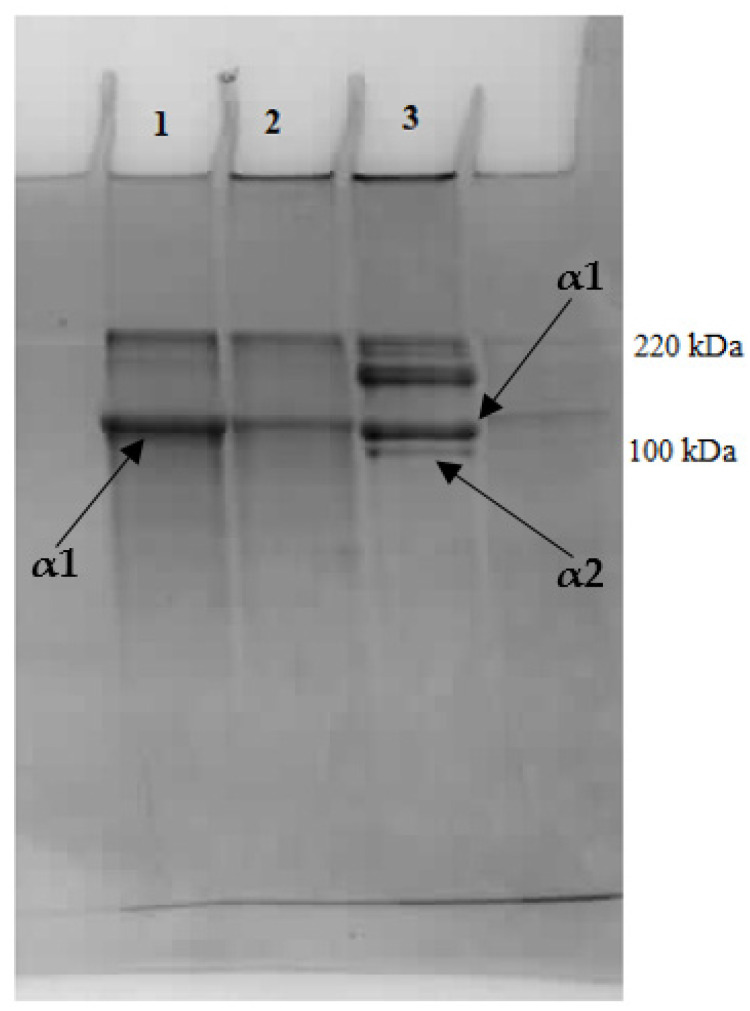
SDS–polyacrylamide gel electrophoresis of atelocollagen fibers isolated from chicken keel cartilage after preliminary proteoglycan extraction with (1) MgCl_2_ solution; (2) NaCl solution; (3) collagen type I (Sigma Aldrich).

It should also be emphasized that the collagen preparations obtained in our research are not completely purified (e.g., GAG residues may reach 2–3%—Table 2), which, as Stamov et al. [39] pointed out, may already affect the structure of collagen fibers. In addition, in the obtained collagen preparations that were characterized by a predominant content of type II, as indicated by the presence of only an α1 chain in the PAGE SDS pattern (Figure 6) (unlike the α1 and α2 chain bands for the collagen type I Sigma standard), it may be contaminated to a small extent with other types of collagen. This may affect the morphology of the reconstituted fibers, including creating FLS fibrous long spacing collagen forms [40], which is shown by banding with periodicity greater than that of native collagen. This can affect the properties of the obtained collagen preparations.

## 3. Materials and Methods

### 3.1. Materials

The material used for this study was chicken keel cartilage, which was obtained from the broiler chicken carcass (5-week-old ROSS line), after cutting from the bony parts of the sternum. In young chickens, it is possible to obtain the sternum cartilage without bone contamination, both in the laboratory or even on an industrial scale. The cartilage was mechanically and hand cleaned from the remains of muscle tissue and was ground in a Retsch SM 2000 mill with a mesh diameter of 2 mm (Retsch, Haan, Germany); then, it was frozen and stored at −20 °C for no more than 2 months. Pepsin (400 units/mg protein, Sigma Aldrich, Steinheim, Germany) from the gastric mucosa of pigs was applied.

### 3.2. Extraction of Collagen Type II

#### 3.2.1. Pretreatment of the Raw Material (PG/GAG Extraction)

Proteoglycans were extracted from the raw material using the following solvents:

(a) 0.2 M and 1.0 M NaCl solution in 0.05 M Tris-HCl buffer, pH 7.5, 24 h, 1:8 (*w*/*v*), 4 °C according to Cao and Xu [8],
(b) 3 M MgCl_2_ solution, pH 7.1, 24 h, and 48 h, 1:8 (*w*/*v*), 23 °C [41].

The extract was separated by centrifugation at 15,000× *g*, at 4 °C (15 min). In the supernatant, the amount of hyaluronic acid and glycosaminoglycans was identified to determine the degree of PG extraction. The cartilage after pretreatment with NaCl or MgCl_2_ solution was analyzed for thermal properties using differential scanning calorimetry. Then, the precipitate of connective tissue was digested with pepsin to obtain atelocollagen type II.

#### 3.2.2. Extraction of Pepsin-Solubilized Collagen

The residue of cartilage containing the collagen fraction (after PG/GAG extraction) was washed 4 times with distilled water (1:9 *w*/*v*) to remove residues of salt solutions. Desalinated cartilage tissue was further treated enzymatically with pepsin in citric acid solution, pH 2.0. Extraction of collagen type II was performed with pepsin in the amount of 400 mg/g cartilage in citric acid solution at pH 2.0. Incubation was carried out for 24 h in a water bath with stirring (120 rpm) at 23 °C. The ratio of raw material to solution was 1:10 (*w*/*v*). After the end of the process, the samples were centrifuged (15,000× *g*, 15 min, 4 °C). Then, the reconstitution of collagen fibers was carried out via precipitation (self-assembly) from the solution as a result of increasing the ionic strength. For this purpose, enough NaCl was added to the atelocollagen-containing supernatant to obtained 0.9 M final salt concentration in solution, which was left for 16 h at 4 °C. Then, the solutions containing the precipitated collagen fibers were centrifuged (15,000× *g*, 30 min, 4 °C), and the resulting precipitate was analyzed as “acid fibers” or dialyzed (dialysis membrane with 12,000 Da cut-off) against 0.01 M phosphate buffer solution (pH 7.4) at 4 °C to obtain the “neutral” (pH 7.4) form of collagen fibers [8]. Neutral collagen fibers were tested in wet form or lyophilized to create structures/scaffolds for biomaterials. The purity of type II collagen preparations was verified by SDS-PAGE electrophoresis conducted on 10% gel according to Laemmli [42] using as a standard Sigma Aldrich (Steinheim, Germany, C7661) type I collagen from rat tail. 

### 3.3. Proximate Compositions of Cartilage

Raw materials were analyzed for dry matter, ash, fat, and protein by the standard methods AOAC (2005).

### 3.4. Quantitative Determination of Collagen (Hydroxyproline) and Proteoglycans or Glycosaminoglycans (Hyaluronic Acid and Chondroitin Sulfate) Content

Hydroxyproline (Hyp) was determined using the spectrophotometric method according to ISO 3496: 1994(E), and Hyp content was used to balance collagen yield in individual fractions. To determine the approximate collagen content in raw cartilage, the Hyp content was multiplied by 7.57 (the only indicator reported in the literature for poultry collagen [43]. Hyaluronic acid was determined based on the method reported by Bitter and Muir [44]. The samples were hydrolyzed with 0.025 M sodium tetraborate (Na_2_[B_4_O_5_;(OH)_4_] × 10 H_2_O) in sulfuric acid (100 °C, 10 min). Then, 0.125% solution of carbazole in absolute ethanol was added, and the samples incubated for 15 min in a water bath at 100 °C. The absorbance was measured at a wavelength of 530 nm. Standard curve was prepared for D-(+)-glucuronic acid (concentration from 4 to 40 µg/mL). Glycosaminoglycans were measured spectrometrically (at 525 nm, color reaction with dimethylmethylene blue) according to method described by Farndale et al. [45] using papain for protein hydrolysis. A standard curve was prepared from chondroitin 4-sulfate.

### 3.5. Differential Scanning Calorimetry (DSC)

The thermal properties of cartilage and collagen were analyzed using a differential scanning calorimeter DSC821e (Mettler Toledo, Greifensee, Switzerland). Samples weighing 30–80 mg were placed in medium-pressure stainless steel crucibles (120 μL capacity). Heating was carried out in the temperature range of 15 to 120°C. The heating rate was 0.5 °C/min. Nitrogen (N2 5.0) was used as the shield gas at a flow rate of 60 mL/min. The reference sample was an empty vessel (medium-pressure stainless steel crucible) with a mass similar to that of the vessel with the sample. The temperature of maximum transition T_m_ (the minimum of the heat curve) was recorded from the thermogram, and total denaturation enthalpy (ΔH) was determined by measuring the area of the DSC thermogram and expressed in Joule per gram of cartilage, fibers, or collagen content [J/g].

### 3.6. Rheological Properties of Reconstituted Collagen Fibers

The rheological characteristics of 2% collagen fiber solutions in 0.01 M phosphate buffer solution (pH 7.4) were tested during the heating cycle from 10 to 70 °C and then cooled from 70 to 4 °C at a rate of 1.0 °C/min using Haake RheoStress 6000 oscillating rheometer (Thermo Fisher Scientific, Waltham, MA, USA). Measurements were carried out in the plate-cone system (C60/1°TiL) with a diameter of 35 mm and angle of 1°. Changes in rheological characteristics expressed as values of the storage modulus (G′) were tested at a frequency of 0.05 Hz and oscillating stress of 1.0 Pa.

### 3.7. Scanning Electron Microscopy (SEM)

The microstructure of the analyzed samples was observed using a Tesla BS 300 scanning microscope (TESCAN, Brno, Czech Republic) at an accelerating voltage of 5 kV and magnifications from 100× to 10,000×. Initially, the neutral wet atelocollagen fibers were washed in tert-butyl alcohol to avoid artificial sticking according to the method described by Zhang et al. [25]. Then, reconstituted neutral fibers (prewashed) or their lyophilized preparations were fixed with 2.5% glutaraldehyde (GA) in 0.1 M phosphate buffer (pH 7.6). The next stage of dehydration was conducted using ethanol solutions of increasing concentration (50%, 70%, 80%, 90%, 95%, and 100%), drying at the critical point using CO_2_, and finally spraying the preparation with technical gold.

### 3.8. Statistical Analysis

In the experiments, three batches of cartilage were used, performing three independent extractions and collagen fibrillogenesis for one batch of raw material (3 × 3 = 9). Measurements are presented as means ± standard deviation (SD). Statistical analysis was performed using the STATISTICA 13.3 (StatSoft, Tulsa, OK, USA). The results obtained were analyzed using one-way analysis of variance (ANOVA), and Duncan’s multiple range test was applied to determine the statistically significant differences (a, b, c, d). The tables summarize the mean values and probability (*p*-value) relating to one experimental discriminant for the entire experiment. 

## 4. Conclusions

The type of cartilage pretreatment with salt solutions of different chaotropic properties affects the characteristics of the reconstituted atelocollagen fibers and matrices. The removal of proteoglycans from cartilage with MgCl_2_ with strong chaotropic properties reduces the potential ability of atelocollagen self-assembly in solutions (70% of the total amount of atelocollagen), and the resulting collagen fibers are thicker than those obtained from cartilage after PG removal with NaCl. Thinner collagen fibers show phase transitions, which are analyzed as changes in rheological characteristics (including gelatinization) at higher temperatures and form regular scaffolds after freeze drying, in contrast to thin spatial structures obtained after freeze drying of collagen fibers obtained after pretreatment of cartilage in MgCl_2_ solution. The conducted research allows the recognition of the possibility of creating biomaterials from a new raw material, i.e., waste poultry cartilage, and it extends the knowledge about the structure and properties of reconstituted type II collagen, which has been so far produced only from bone skeletal elements of fish, pigs, and cattle. The obtained preliminary results allow us to plan further research related to various methods of forming biomaterials from the reconstituted poultry cartilage collagen, including the combination of type II collagen matrices with glycosaminoglycans. Special attention will be paid to the effect of small amounts of GAG, which can remain in the collagen preparations after the purification procedure or intentionally added to the matrices.

## Figures and Tables

**Table 1:** The chemical composition of the chicken keel cartilage.

Parameters	Concentration [%]	
	Raw Tissue ± SD	in Dry Matter
dry matter	13.10 ± 0.81	100.00
protein	8.50 ± 0.26	64.30
ash	0.90 ± 0.10	7.00
fat	0.09 ± 0.02	0.67
hydroxyproline	0.95 ± 0.13	7.25
collagen	7.19 ± 0.98	54.40
uronic acid	1.40 ± 0.34	10.70
glycosaminoglycans	3.80 ± 0.42	28.80

**Table 2:** The extraction yield of uronic acid (UA) and glycosaminoglycans (GAG).

Extraction Conditions			Yield [%]	
Type of Salt Solution	Time [h]	Temperature [°C]	UA	GAG
0.2/1.0 M NaCl	24	4	97.3	98.5
3M MgCl_2_	24	23	85.6	83.8
	48		97.9	98.0
*p*-Value			0.48	0.38

**Table 3:** Characteristics of the reconstituted and freeze-dried collagen fibers.

Type of Material after Pretreatment	Yield of Collagen Recovery from Cartilage [%]	Recovery of Collagen in the Form of Fibers [%]	Amount of Freeze-Dried Collagen Fibers [mg/g Cartilage]
0.2 /1.0 M NaCl	95.1	95.2 ^a^	120
3 M MgCl_2_	97.3	70.8 ^b^	100
*p*-Value	0.33	0.02	0.29

^a,b^*—*the statistically significant differences with *p* ≤ 0.05.

**Table 4:** Thermal characteristics of cartilage and collagen fibers determined by scanning calorimetry.

Material	T_m_ [°C]	ΔH [J/g Cartilage/Fibers]	ΔH [J/g Collagen]
Chicken keel cartilage	64.5	0.5	7.1
Cartilage residue after treatment in MgCl_2_ solution	62.5	1.8	17.7
Cartilage residue after treatment in NaCl solution	65.0	2.1	23.3
Collagen fibers MgCl_2_ (acidic)	45.5	4.3	118
Collagen fibers NaCl (acidic)	46.1	4.3	96.4
Collagen fibers MgCl_2_, neutral pH	55.9	5.1	219
Collagen fibers NaCl, neutral pH	55.8	3.6	156

T_m_—maximum transition temperature, ΔH—total denaturation enthalpy.

## Data Availability

Data are available from the corresponding author.

## Sample Availability

Samples are available from the authors.
